# New Hydrogels Nanocomposites Based on Chitosan, 2-Formylphenylboronic Acid, and ZnO Nanoparticles as Promising Disinfectants for Duodenoscopes Reprocessing

**DOI:** 10.3390/polym15122669

**Published:** 2023-06-13

**Authors:** Daniela Ailincai, Ioana-Andreea Turin Moleavin, Alexandra Sarghi, Adrian Fifere, Oana Dumbrava, Mariana Pinteala, Gheorghe G. Balan, Irina Rosca

**Affiliations:** 1 “Petru Poni” Institute of Macromolecular Chemistry, 700487 Iasi, Romania; ailincai.daniela@icmpp.ro (D.A.); moleavin.ioana@icmpp.ro (I.-A.T.M.); iacobescu.alexandra@icmpp.ro (A.S.); fifere@icmpp.ro (A.F.); dumbrava.oana@icmpp.ro (O.D.); pinteala@icmpp.ro (M.P.); 2 Faculty of Medicine, ‘Grigore T. Popa’ University of Medicine, 700115 Iasi, Romania; balan.gheo@me.com; 3 Institute of Gastroenterology and Hepatology, St. Spiridon Emergency County Hospital, 700111 Iasi, Romania

**Keywords:** chitosan hydrogels, boronic aldehyde, ZnO, disinfectant

## Abstract

New hydrogels nanocomposites, based on iminoboronate hydrogels and ZnO nanoparticles (ZnO–NPs), were obtained and characterised in order to develop a new class of disinfectants able to fight the nosocomial infections produced by duodenoscopes investigation procedures. The formation of the imine linkages between chitosan and the aldehyde was demonstrated using NMR and FTIR spectroscopy, while the supramolecular architecture of the developed systems was evaluated via wide-angle X-ray diffraction and polarised optical microscopy. The morphological characterisation of the systems via scanning electron microscopy revealed the highly porous structure of the materials, in which no ZnO agglomeration could be observed, indicating the very fine and homogenous encapsulation of the nanoparticles into the hydrogels. The newly synthetised hydrogels nanocomposites was proven to have synergistic antimicrobial properties, being very efficient as disinfectants against reference strains as: *Enterococcus faecalis*, *Klebsiella pneumoniae*, and *Candida albicans*.

## 1. Introduction

One of the most concerning outcomes of recent years has been the nosocomial infection outbreaks brought on by multidrug-resistant organisms (MDRO) after endoscopic retrograde cholangiopancreatography (ERCP) procedures [1]. Endoscopic retrograde cholangiopancreatography (ERCP), first performed in 1968, is an advanced medical treatment designed to perform a minimally invasive diagnosis and treat pancreaticobiliary disorders [2,3]. Due to their intricate construction, duodenoscopes are challenging for proper reprocessing [4,5,6].

After reprocessing, duodenoscope contamination rates range from 0.4% to 35.8% [1,7,8,9,10,11,12,13]. The majority of nosocomial infections display adhesion problems at duodenoscopes, making it challenging to access these surfaces for reprocessing due to their persistence. The most frequently encountered pathogens, such as *Klebsiella pneumoniae*, *Pseudomonas aeruginosa*, *Escherichia coli*, and *Salmonella faecalis*, are known for their ability to form an MDRO and adhere to surfaces and spaces that are difficult to access for reprocessing [14,15,16,17,18]. Our team has prior research experience in this area, with studies describing the effects of routine procedure use and reprocessing cycles on the duodenoscope, demonstrating usage-related changes to both coating and working channel polymers, and demonstrating that the external changes progress progressively from the distal to the proximal to the elevator sample [19,20]. According to reported duodenoscope contamination rates, present reprocessing techniques are insufficient. This highlights the need for additional research into novel classes of disinfectants to enhance reprocessing outcomes and prevent future outbreaks and infections linked to duodenoscopy.

Within this framework, the literature abounds in information related to the antimicrobial activity of ZnO–NPs against *Escherichia coli* [21,22,23], *Pseudomonas aeruginosa* [24], *Staphylococcus aureus* [25,26], *Klebsiella pneumonia* [22,27], *Enterococcus faecalis* [28,29,30,31], and *Candida albicans* [29,32]. ZnO–NPs activity against drug-resistant pathogens is considered to depend on size and doping amount; they can be cost-effective and are quite stable for long-term storage with a prolonged shelf-life [33]. Moreover, MeO–NPs can be subjected to sterilisation via methods of high temperature, gamma irradiation, or plasma treatment without losing their properties or inactivation [34]. In the future, they could be combined with various classes of substances for optimal antimicrobial activity due to their additive nature [35].

Within this context, this study aims to obtain a new class of disinfectants based on the synergic activity of chitosan, boronic aldehyde, and ZnO and able to surpass the difficulties of duodenoscope reprocessing and to decrease the incidence of nosocomial infections in this procedure. Previous studies on iminoboronate chitosan hydrogels demonstrated the very high antifungal activity of these systems, on both planktonic yeast and biofilm [36] on two *Candida* strains—*C. glabrata* and *C. albicans*. Moreover, hydrogels based on chitooligosaccharides and boronic aldehyde presented high activity against nine reference strains: *Staphylococcus aureus*, *Escherichia coli*, *Candida albicans*, *Candida parapsilosis*, *Candida glabrata*, *Saccharomyces cervisiae*, *Penicillium crysogenum*, *Cladosporium cladosporioides*, and *Aspergillus brasiliensis* [37]. ZnO–NPs, on the other hand, are known in the literature for their antimicrobial properties against various microbial strains [38].

The aim of the present study is to obtain new disinfectants in the form of hydrogels based on iminoboronate chitosan derivatives, in which the ZnO–NPs should be homogenously dispersed, with the goal of both reaching synergism and broadening the antimicrobial activity of the hydrogels to a large number of microorganisms through the presence of ZnO–NPs. This combination has not been reported elsewhere, as far as we know.

## 2. Materials and Methods

### 2.1. Materials

Chitosan (147 kDa, DD = 88%), 2-formylphenylboronic acid (2-FPBA), 95% zinc acetate dihydrate, sodium hydroxide, acetic acid, and ethanol were purchased from Sigma (St. Louis, MO, USA) and used as received.

### 2.2. The Synthesis of ZnO Nanoparticles (ZnO–NPs)

The synthesis of ZnO–NPs was performed following a previously reported study, using the co-precipitation method [39]. Briefly, 30 mL Zn(Ac)_2_·2H_2_O (0.1 M) solution and 25 mL NaOH (0.2 M) solution in H_2_O were subjected to magnetic stirring for 2 h at a temperature of 60 °C. After 2 h, the initially clear solution turned into a milky white dispersion. The resulting white product was separated via centrifugation at 5000 rpm for 10 min and washed 3 times with ultrapure water. ZnO–NPs, in powdered form, were obtained by drying the product in a conventional laboratory oven at 35 °C for 48 h.

### 2.3. The Synthesis of the Hydrogels Containing ZnO–NPs

Three hydrogels containing ZnO–NPs were synthesised using an in situ hydrogelation method of chitosan with 2-formylphenylboronic acid in the presence of ZnO–NPs. The hydrogels contained the same amount of ZnO but had different crosslinking degrees, depending on the molar ratio used between chitosan and 2-FPBA, ranging from 1/1 to 2/1. At lower molar ratios between the reagents’ functionalities, the hydrogelation was not reached. For example, the experimental protocol for the obtaining of the S2 sample was as follows: to a solution of chitosan in acetic acid aqueous solution (see Table 1), a mixture of ZnO and 2-FPBA in ethanol was added at 55 °C under continuous vigorous magnetic stirring (1500 rot/min). The hydrogelation was reached after different time intervals, depending on the NH_2_/CHO molar ratio, from 5 min for sample S1 to 3 h for samples S2. A sample containing only ZnO dispersed into chitosan was also obtained and used as reference (R).

### 2.4. Characterisation

#### 2.4.1. Hydrogels’ Lyophilisation

The hydrogels containing ZnO–NPs (S1–S2) and the reference sample (R) were freeze-dried using a LABCONCO FreeZone FreezeDry System (Fort Scott, Kansas City, MO, USA), at −54 °C and 1.510 mbar, for 24 h.

#### 2.4.2. FTIR Spectroscopy

The ATR–FTIR spectra of the hydrogels after lyophilisation (xerogels) were recorded on a FTIR Bruker Vertex 70 Spectrophotometer (Ettligen, Germany), with a ZnSe single reflection ATR accessory.

#### 2.4.3. NMR Spectroscopy

The ^1^H NMR spectra were recorded on a Bruker Avance NEO 400 MHz spectrometer (Ettlingen, Germany, Bruker Biospin) equipped with a 5 mm broadband inverse detection z-gradient probe, using NOESY water presaturation–pulse sequence. All the spectra were recorded through 64 scans and were manually processed with Bruker TopSpin 4.2.0 spectrometer control and processing software.

#### 2.4.4. Scanning Electron Microscopy

The morphology of the xerogels was investigated with a Scanning Electron Microscope SEM EDAX—Quanta 200 (Eindhoven, Germany) and the obtained images were processed with Image J and Origin Pro 8 Software.

#### 2.4.5. Wide-Angle X-ray Diffraction

Wide-angle X-ray diffraction analysis was performed on a Rigaku Miniflex 600 diffractometer using CuKα emissions in the angular range of 2–80° (2θ) with a scanning step of 0.0025° and a recording rate of 1°/min.

#### 2.4.6. Polarised Optical Microscopy

The obtained systems were evaluated through polarised optical microscopy (POM) on a Leica DM2500 microscope (Hamburg, Germany).

#### 2.4.7. Atomic Force Spectroscopy (AFM)

The samples collected from surface of the duodenoscope and the samples exposed to the hydrogel and microbial strains were analysed using an NTEGRA Spectra (NT-MDT, Zelenograd, Russia) instrument with the 3.1–37.6 N/m force constant cantilever of a silicon nitride cantilever (NSC10; NT-MDT, Russia) in tapping mode.

### 2.5. Antioxidant Activity

The antioxidant activity of the samples was evaluated by analysing their ability to capture the 2,2-diphenyl-1-picrylhydrazyl (DPPH) radicals from a stock solution 0.025 mg DPPH/ mL in ethanol. The spectra of all samples, DPPH control solution, and the solutions of the samples, incubated with the DPPH solution, were recorded using an UV–Vis Spectrophotometer Cary 60. The DPPH maximum from 517 nm was used for the calculation of the samples’ radical scavenging ability. Known amounts of samples, all containing 10 mg of chitosan, were dissolved in 1.5 mL water and 10.5 μL acetic acid. To these solutions, 1.5 mL DPPH solution in ethanol was added and incubated for 1 h at room temperature and in the dark. After 1 h, the UV–VIS spectra were recorded and the absorbance at 517 nm was read.

The antioxidant activity of the samples (RSA%) was calculated using the following equation:

$$\mathrm{RSA}=\;\frac{A_{\mathrm{ref}}-A_{\mathrm{sample}}}{A_{\mathrm{ref}}}\times 100,$$

where RSA is the radical scavenging ability, A_ref_ is the absorbance of the DPPH solution without sample, and A_sample_ is the absorbance of the DPPH solution, which was incubated with the samples for 1 h.

### 2.6. Antimicrobial Activity

External samples of 1 cm^2^ each were taken from an international market reference duodenoscope (TJF-160F, Olympus Corporation, Tokyo, Japan), selected from a high-volume tertiary gastroenterology centre, previously used in approximately 500 ERCPs. This model, belonging to the penultimate generation of duodenoscopes, has established itself as one of the most used instruments globally, characterised by reliability over time and high-level technological quality.

The antimicrobial activity screening of the hydrogels samples (S1, S1.5, and S2) and controls (chitosan as Ch; ZnO,2-formylphenylboronic acid as BA; and chitosan + ZnO as sample R) was performed by using viable cell-counting method [40]. Three reference strains with high incidence in the nosocomial infections produced by ERCPs were used, i.e., *Staphylococcus aureus* ATCC25923 (*S. aureus*), *Klebsiella pneumoniae* ATCC10031 (*K. pneumoniae*), and *Candida albicans* ATCC90028 (*C. albicans*). The bacterial strains were refreshed on nutrient agar (NA) and the yeast strain was refreshed on Sabouraud dextrose agar (SDA) at 37 °C. Microbial suspensions of these cultures were prepared in sterile nutrient broth medium in order to obtain turbidity optically comparable to a 0.5 of the McFarland standards. A total of 500 μL of the hydrogels and controls was placed into the solution, which contained 0.5 mL bacterial suspension and 4.5 mL 1X PBS solution as well as the coating samples for the duodenoscopes, followed by incubation in a shaker at 37 °C up to 24 h. A control experiment was conducted. A total of 5 μL of the control samples and of the treated samples was removed at determined periods of incubation time (10 min, 20 min, 40 min, 1 h, 2 h, 3 h, 6 h, and 24 h) and spread on plate count agar (PCA) plates. The number of colonies was counted after 24 h of incubation at 37 °C.

All tests were carried out in triplicate to verify the results. After incubation, the plates were analysed using SCAN1200^®^, version 8.6.10.0 (Interscience, Saint-Nom-la-Bretèche, France). The ratio of the inhibited the growth of bacteria (IRG) was calculated and is defined as:

IRG (%) = (Na − Nb)/Na × 100%

where Na and Nb are the average values of colonies of the control group and the experimental groups, respectively.

The duodenoscope samples were removed, washed with sterile water, and the surface morphology was examined by using a scanning electron microscope SEM EDAX—Quanta 200 (Eindhoven, Germany) and atomic force spectroscopy (AFM) NTEGRA Spectra (NT-MDT, Zelenograd, Russia).

### 2.7. Statistical Analysis

Data analysis was performed using GraphPad Prism software version 7.00 for Windows (GraphPad Software, La Jolla, San Diego, CA, USA, https://www.graphpad.com/, accessed on 1 May 2023). The obtained results represent the mean ± standard deviation (SD) of three different experiments. Two-way ANOVA was applied to determine the statistical significance between the mean of cells viability. A value of *p* < 0.05 was considered statistically significant, and the significance is noted as * *p* < 0.05; ** *p* < 0.01; and *** *p* < 0.001.

## 3. Results and Discussion

**Scheme 1:**
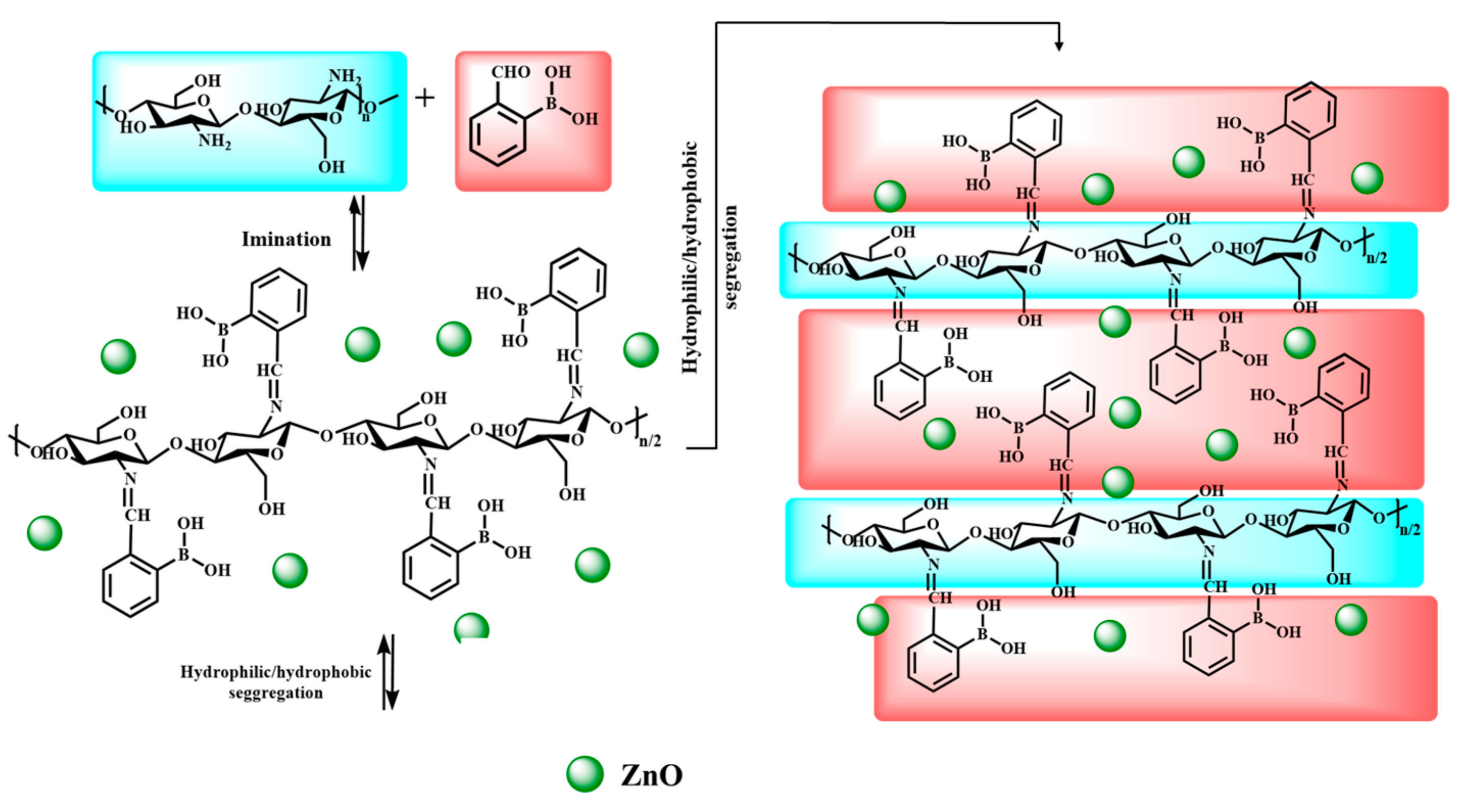
The synthesis of hydrogels via chitosan imination and hydrophilic/hydrophobic segregation in the presence of ZnO.

New hydrogels composites containing ZnO–NPs were obtained through the combination of chitosan with 2-formylphenylboronic acid in three molar ratios of their functionalities (Table 1, Scheme 1). The design of the systems took into consideration the antimicrobial properties of all components in order to obtain materials with strong antimicrobial activity against different strains, usually associated with nosocomial infection outbreaks brought on by MDRO after ERCP procedures. The systems are based on reversible imine linkages, which are kept together in a three-dimensional network by hydrophobic clusters formed through hydrophilic/hydrophobic segregation, forming a matrix in which ZnO–NPs can be obtained [37,41,42].

### 3.1. Structural Characterisation by ^1^H-NMR and FTIR Spectroscopy

**Figure 1:**
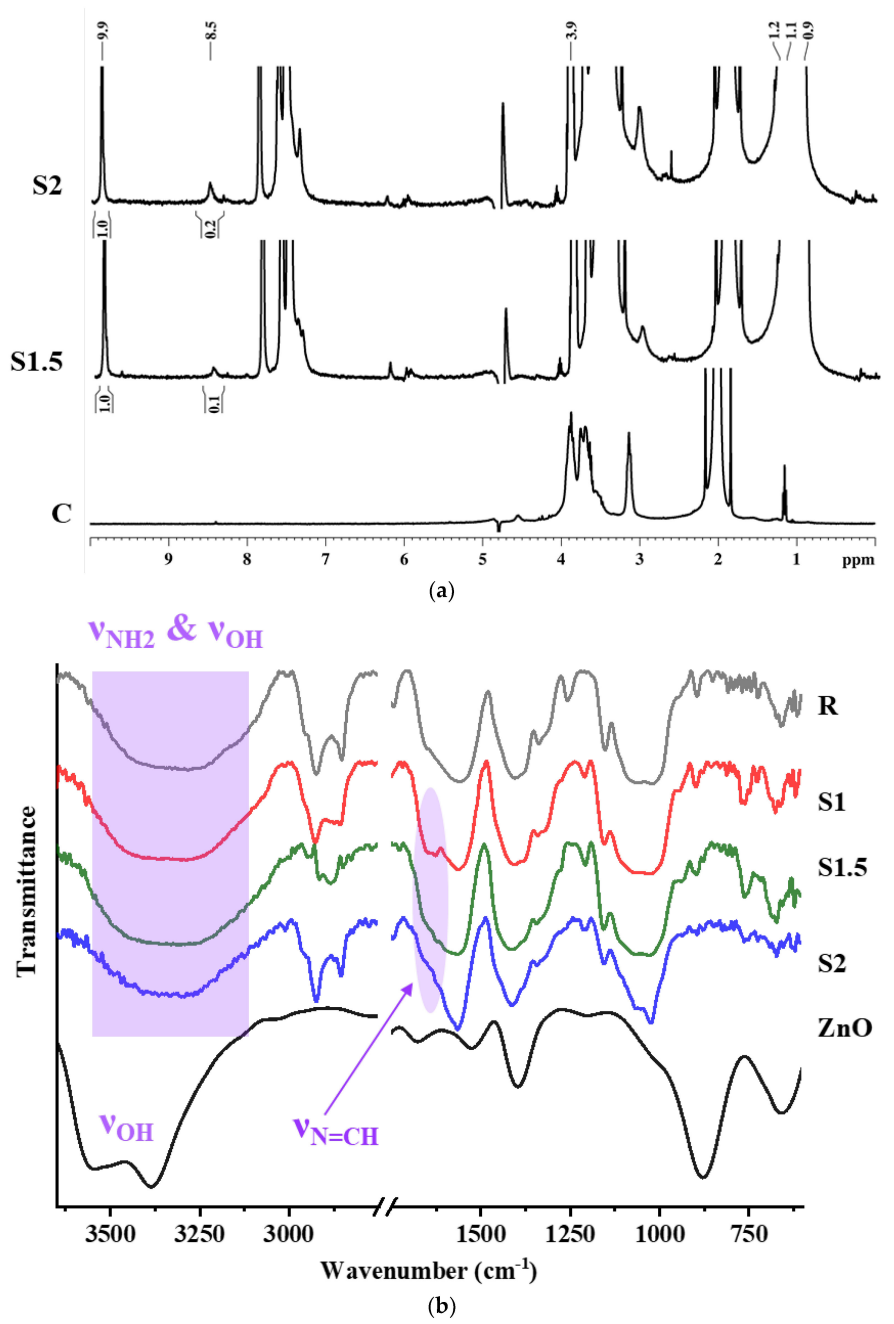
NMR spectra of representative samples (**a**) and FTIR spectra of the prepared samples (**b**).

The structural characterisation of the hydrogels was first carried out using ^1^H-NMR spectroscopy, through comparison with chitosan. The chitosan spectrum presented the characteristic chemical shifts according to its chemical structure: at 3.1 ppm, the H2 from the D-glucoamine unit appeared, and between 3.4 and 4.2 ppm, superposed signals corresponding to the H3–H6 protons from the D-glucosamine unit appeared. Due to the water suppression, the H1 signal was not present in the spectrum (Figure 1a). In the spectra of the S1.5 and S2 hydrogels, besides the signals from chitosan, a new peak appeared at 8.5 ppm, corresponding to the imine proton. Moreover, the chemical shift corresponding of the unreacted aldehyde was also present as an intense peak at 9.9 ppm. This can be explained by the fact that the imine formation is an equilibrium reaction which depends mainly on the aldehyde’s reactivity. Previous studies on iminoboronate chitosan hydrogels demonstrated the farily low reactivity of 2-FPBA, indicating its incomplete consumption in the imination reaction with chitosan. In this particular case, very probably because of the presence of ZnO–NPs, the imine formation was hampered, with the imine to aldehyde integrals ratio being lower than in the absence of ZnO–NPs [36,37].

FTIR spectroscopy was used as a complementary method for the structural characterisation of the obtained systems. The FTIR spectrum of the ZnO–NPs presented a similar pattern to those found in the literature, and the bands that appeared in the finger print region, between 1400 and 600 cm^−1^, were characteristic to ZnO [43]. Moreover, the broad band with two maxima at 3545 and 3385 cm^−1^ (Figure 1) represents the OH stretching vibrations due to the absorbed water molecules on the surface of ZnO–NPs. Regarding the FTIR spectrum of the R sample, it presents some differences in terms of bands positions and intensities, compared to those in ZnO, indicating strong interactions between chitosan and the nanoparticles. The structural characterisation of the xerogels by FTIR revealed a new band at 1625 cm^−1^, indicating the formation of imine linkages between chitosan and boronic aldehyde. The band is broad due to being superposed with the amide band from chitosan [36]. The changes that appeared in the 3700–2700 cm^−1^ spectral region are due to the fact that the signals are shifted to lower wavenumber as a consequence of chitosan’s presence, and the band in that region corresponds to the amino and hydroxyl stretching vibrations from its structure [44].

FTIR spectroscopy was used as a complementary method for the structural characterisation of the obtained systems. The FTIR spectrum of the ZnO–NPs presented a similar pattern to those found in the literature, and the bands that appeared in the finger print region, between 1400 and 600 cm^−1^, were characteristic to ZnO [43]. Moreover, the broad band with two maxima at 3545 and 3385 cm^−1^ (Figure 1) represents the OH stretching vibrations due to the absorbed water molecules on the surface of ZnO–NPs. Regarding the FTIR spectrum of the R sample, it presents some differences in terms of bands positions and intensities, compared to those in ZnO, indicating strong interactions between chitosan and the nanoparticles. The structural characterisation of the xerogels by FTIR revealed a new band at 1625 cm^−1^, indicating the formation of imine linkages between chitosan and boronic aldehyde. The band is broad due to being superposed with the amide band from chitosan [36]. The changes that appeared in the 3700–2700 cm^−1^ spectral region are due to the fact that the signals are shifted to lower wavenumber as a consequence of chitosan’s presence, and the band in that region corresponds to the amino and hydroxyl stretching vibrations from its structure [44].

### 3.2. Supramolecular Characterisation via Wide-Angle X-ray Diffraction

**Figure 2:**
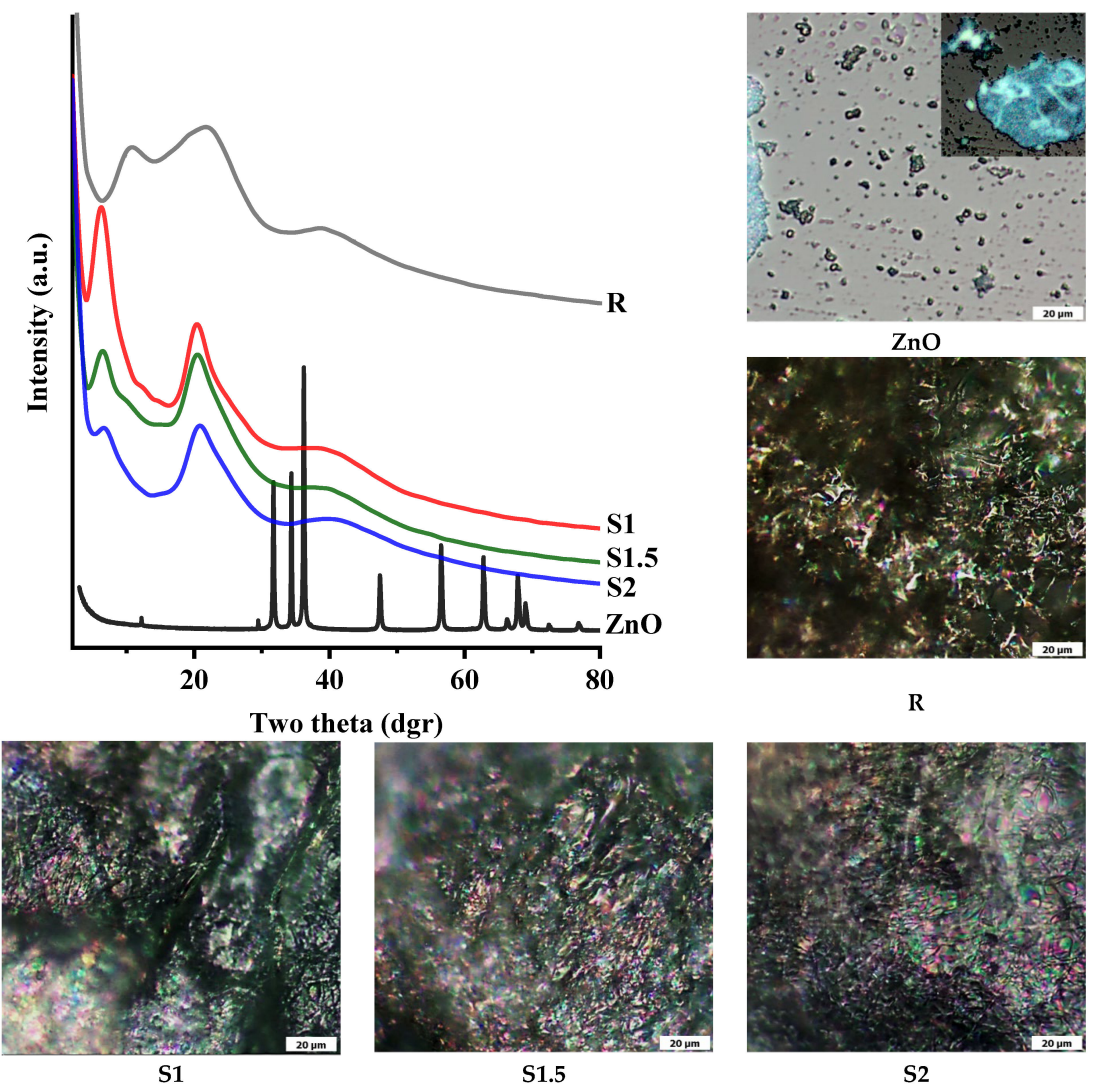
X-ray diffractograms of the prepared samples and polarised optical microscopy images.

The supramolecular arrangement of the imino-chitosan xerogels containing ZnO was investigated via WXRD (see Figure 2), through comparison with the reference samples, ZnO, and R. The pattern of the ZnO is similar to that found in the literature, indicating the high purity of the obtained nanoparticles [45], indexed within the hexagonal ZnO wurtzite, according to Joint Committee on Powder Diffraction Standards (JCPDS) card no. 36–1451 [46]. The diffraction peak from 36.2 two theta degrees was used in order to evaluate the size of the crystallites by applying the Debye–Scherrer Equation [45]:

$$D=\frac{K\times \lambda }{\beta \times \cos\theta }\;,$$

where D is the NP crystalline size, K is the Scherrer constant (0.98), λ is the wavelength of Copper-K radiation (1.5406 Å), and β represents the full width at the half maximum (FWHM) of the diffraction peak. The average size of the ZnO–NPs was determined to be 78 nm.

The diffractogram of the R sample presented three broad diffraction peaks, at 10.7, 22, and 39 two theta degrees, corresponding to the distances of 8.2, 4.1, and 2.4 Å. The first two peaks are from chitosan, while the last peak is from ZnO, being more shifted than the signals of the pure ZnO, indicating significant changes in the oxide supramolecular architecture, due to their interactions with the chitosan backbone. The diffractograms of the xerogels containing ZnO presented a similar pattern with previously reported xerogels based on iminoboronate chitosan derivatives [41], with a difference that also appeared in R’s diffractogram: a sign of the presence of ZnO in the hydrogel matrix.

The high degree of ordering of the samples was also confirmed through POM (Figure 2). The ZnO–NPs, due to their small size, were not clearly observed with the optical microscope, but their agglomeration caused birefringence due to their crystalline nature (Figure 2). All the other samples presented strong birefringence under polarised light due to their highly ordered structure at a supramolecular level, as was also evidenced by XRD.

### 3.3. Morphological and Elementary Characterisation by SEM and EDAX

**Figure 3:**
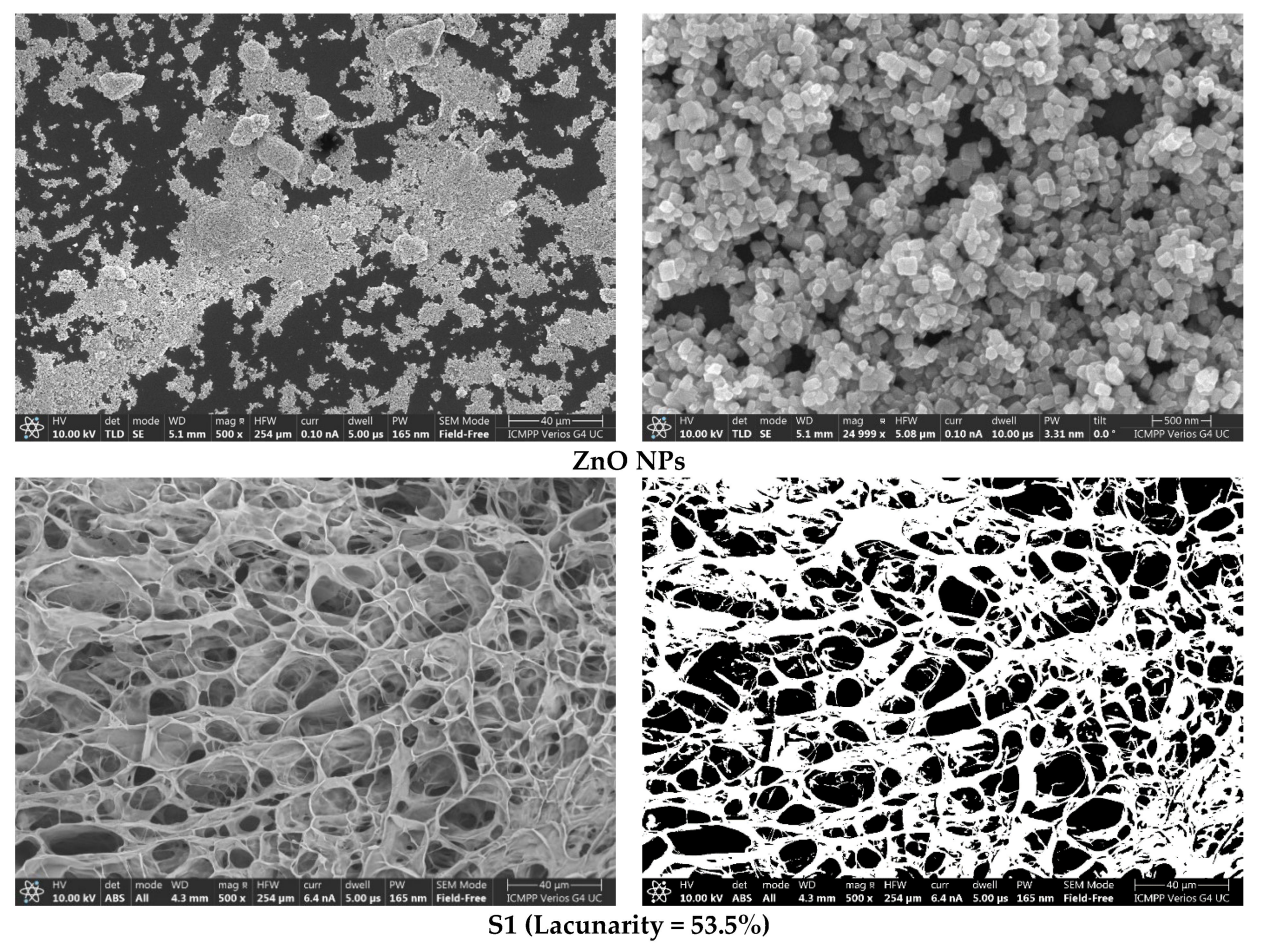

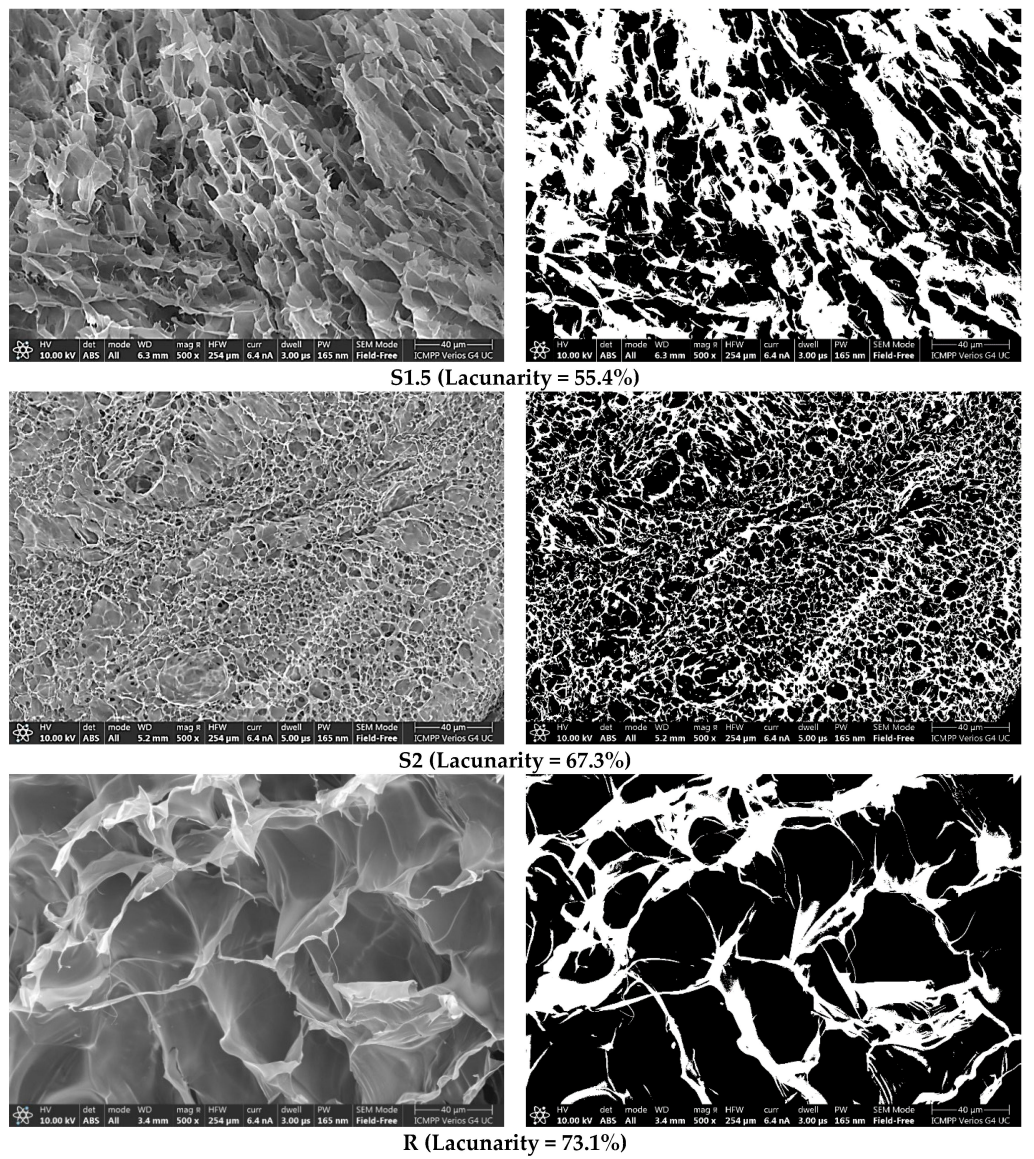
SEM microphotographs and 8-bit images obtained on the investigated samples.

The morphology of the samples was investigated via scanning electron microscopy. The ZnO powder was also investigated, confirming that nanoentities with regular shape and a mean diameter of ~80 nm were obtained. All the samples presented a highly porous microstructure with pores of micrometric sizes. A porous morphology was also obtained for the reference sample (R) by adding ZnO to the chitosan solution without using 2-FPBA for hydrogelation. This sample had the largest pores, around 40 μm, and a lacunarity of 73%. In comparison with the reference sample, the hydrogels containing ZnO had much lower pore sizes, around 10 μm, and a lacunarity that was dependent on the aldehyde content from 53% for S1 to 67% for S2. Another important observation after SEM investigation was the fact that no ZnO aggregates were observed in the microphotographs confirming the very fine distribution of the ZnO into the hydrogels (Figure 3).

**Figure 4:**
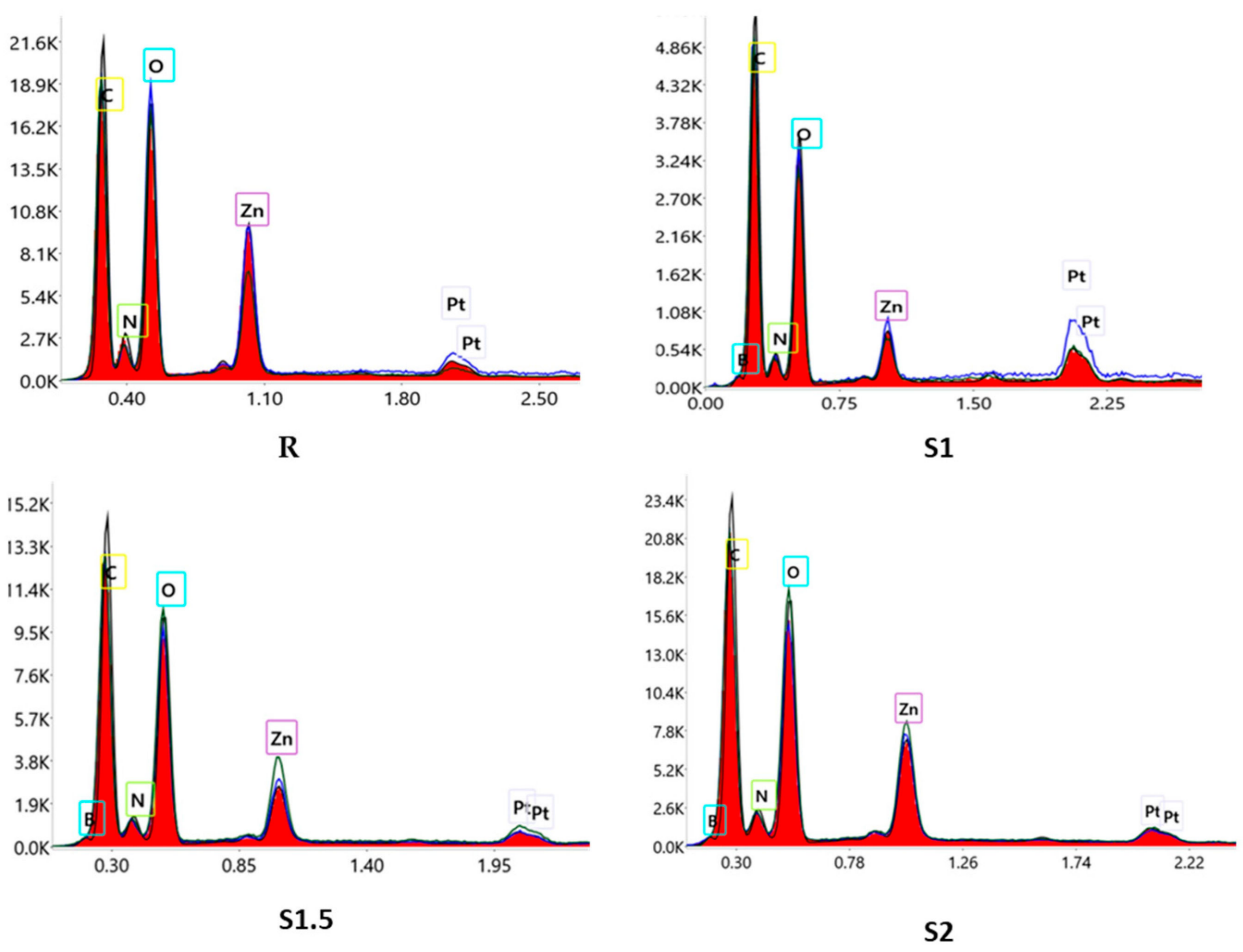
EDAX spectra for the samples, evidencing the presence of ZnO in the systems.

To attest the presence of the ZnO in the hydrogels, the samples were submitted to energy-dispersive X-ray analysis (EDAX). As can be observed, in the R sample, containing chitosan and ZnO as well as the chemical elements from chitosan (C, H and N), Zn also appeared, due to the presence of ZnO. The same also occurred in the case of the hydrogels, where, alongside the elements from chitosan and 2-FPBA (C, O, and B), zinc also appeared (Figure 4).

### 3.4. Antioxidant Activity

**Figure 5:**
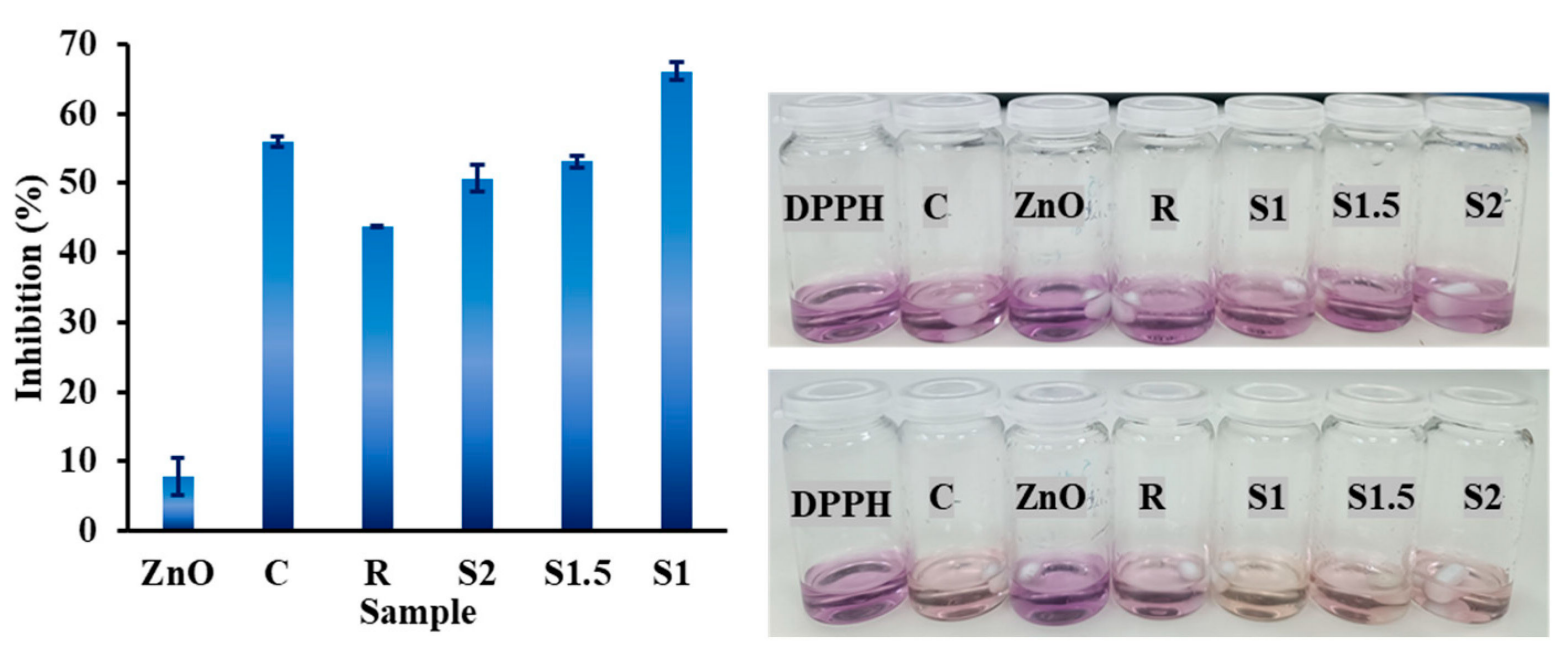
The antioxidant activity of the hydrogels against 1,1-diphenyl-2-picryhydrazyl (DPPH) and pictures before and after sample incubation with DPPH solution.

The antioxidant activity of the developed hydrogel composites was evaluated using the DPPH assay and compared with those of the reference samples, namely ZnO–NPs, chitosan, and a chitosan–ZnO mixture (Figure 5). The experiments were carried out on the reference samples at the same concentration as those that were used in the formulations. As can be observed from the experimental results, the ZnO–NPs presented a very weak inhibition ability of only ~8%, very probably due to the very low concentration, while chitosan presented a quite high radical scavenging capacity of ~56%. The combination of chitosan with ZnO–NPs (R) presented an intermediate value of 44%, indicating interactions between the components which prevented chitosan from exerting its full antioxidant potential. However, the formulations, based on iminoboronate hydrogels and ZnO–NPs, presented a high antioxidant activity, with a maximum of 66% for the sample S1. The colour of the DPPH solution changed in the presence of the analysed samples from deep purple to yellow/colourless/light purple, the colour shift being directly correlated with the antioxidant capacity of each sample (Figure 5).

All these data show that the hydrogels present a fairly high radical scavenging potential, which may play an important role in the antimicrobial bioapplications of the developed systems.

### 3.5. Antimicrobial Activity

**Figure 6:**
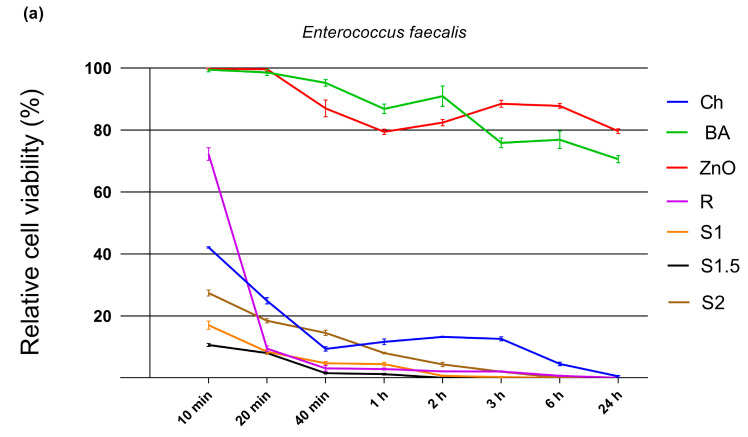

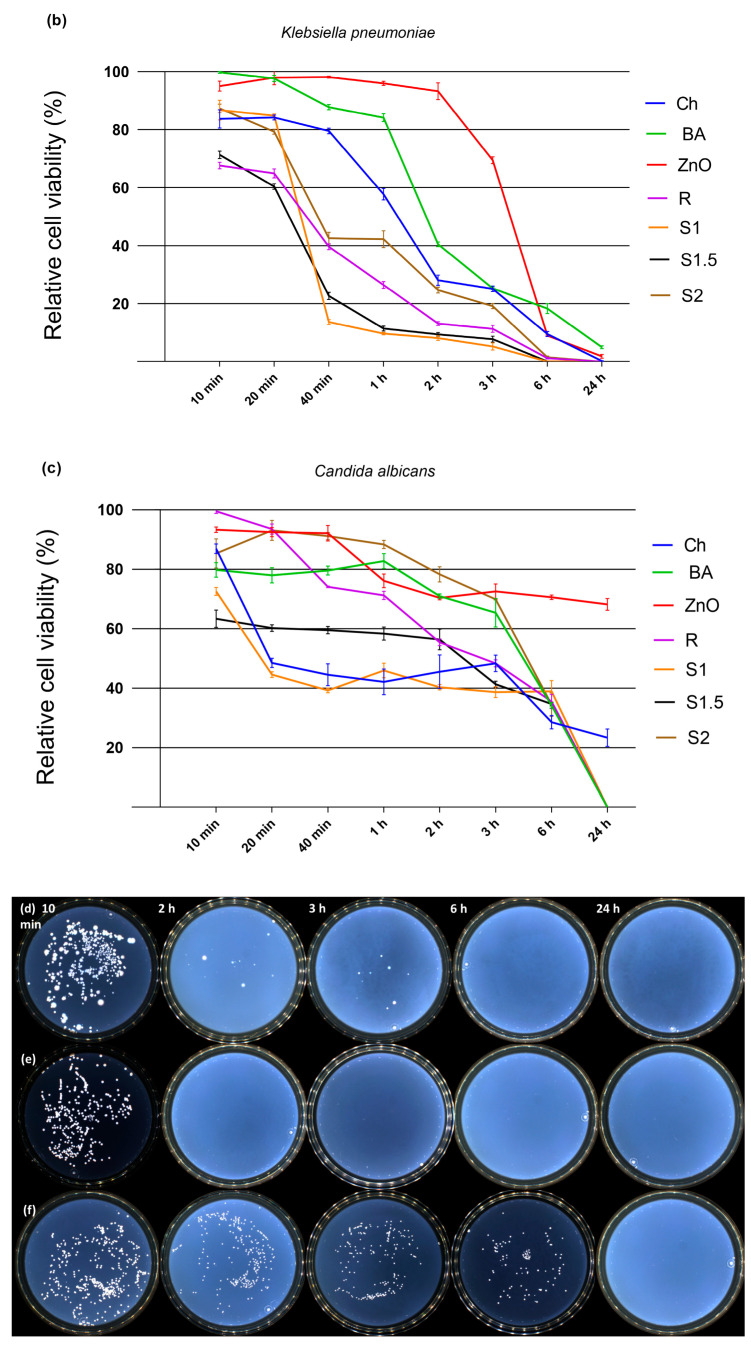

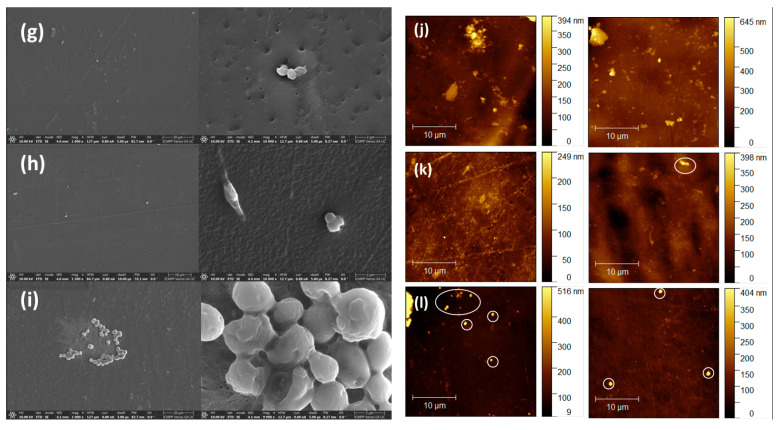
The growth inhibition of the microorganisms, namely (**a**) *E. faecalis*, (**b**) *K. pneumoniae*, and (**c**) *C. albicans*, expressed as percent of viable microorganisms vs. incubation time.; images of (**d**) *K. pneumoniae* after hours of incubation with sample S1; (**e**) images of *E. faecalis* colonies after 24 h of incubation with sample S1.5; (**f**) images of *C. albicans* colonies after 24 h of incubation with sample S1; (**g**) SEM images of the duodenoscope surface after incubation with *E. faecalis* and hydrogel S1; (**h**) SEM images of the duodenoscope surface after incubation with *K. pneumoniae* and hydrogel S1; (**i**) SEM images of the duodenoscope surface after incubation with *C. albicans* and hydrogel S1; (**j**) AFM images of the duodenoscope sample used as a control; (**k**) AFM images of the duodenoscope sample incubated with *K. pneumoniae* and hydrogel S1; (**l**) AFM images of the duodenoscope sample incubated with *E. faecalis* and hydrogel S1.

The diagrams (Figure 6a–c) show the growth inhibition of the microorganism’s colonies when treated with the hydrogel samples and with the controls. Distinct activities against the reference tested strains were noticed (Figure 6d–f). In case of the Gram-positive bacterial strain *E. faecalis*, the cell viability decreased to 0% after 2 h of incubation for samples S1 and S1.5 and to 4% for sample S2 (Figure 6a). In case of the controls, a very reduced antibacterial activity of ZnO and BA was noticed, but an increased activity in the case of Ch and R leading to a synergism was observed in the case of the hydrogels. This type of synergism was noticed for all the tested microorganism strains.

In the case of the Gram-negative bacterial colonies of *K. pneumoniae*, it is very clear that the controls collaborate to obtain the antimicrobial activity of the three types of hydrogels. For sample S1 and S1.5, it took 6 h for all the bacterial cells to be destroyed, while after the same period of incubation, sample S2 reduced the bacterial populations to almost 1%. In this particular case, R also has a very good antibacterial activity, destroying up to 98% of the cells after 6 h of incubation. ZnO and Ch also display notable activity, reducing cell viability by 90% in 6 h.

In the third case, the yeast cells are completely destroyed by the hydrogels after 24 h of incubation. The same activity was also noticed in controls, i.e., BA and R, leading to the same synergism that was previously observed. ZnO had smaller antifungal activity, and Ch was proven to provide an important contribution to the results, destroying up to 75% of the yeast cells after 24 h of incubation.

Microbial cell viability decreased for all the samples in a time-dependent manner. Significantly different cell viabilities among the time points were found (detailed statistics can be seen in Table 2a–c). These results suggested that the microbial cells were damaged by hydrogels and that their toxicity was time-dependent. It was determined that the optimal period of time for the present study is 6 h. According to the two-way ANOVA, a statistically significant (*p* < 0.0001) difference was found between the species when cell viability was considered. Furthermore, the process included the evaluation of the influence of the type of sample and incubation time on the viability or cells. The most significant effect on the cell viability was produced by the incubation time, whereas the material type had less effect.

From the SEM images (Figure 6g–i) and AFM images (Figure 6j–l), it is obvious that the coating material of the duodenoscope exhibits degradation and erosion marks brought on by heavy use and frequent reprocessing. However, the material preserves its anti-adherence properties, but a few very damaged bacterial cells of *E. faecalis* (Figure 6f), *K. pneumoniae* (Figure 6g), and *C. albicans* (Figure 6i) were observed on the tested surfaces after the incubation with the hydrogels, especially with S1, which had the strongest antimicrobial activity of all the tested microbial strains. The same results are also presented by the AFM images, showing that after the incubation of hydrogel S1, there were only a few cells left on the surface of the material, with the hydrogels preventing cell adhesion and biofilm formation.

Figure 6j–l shows the AFM topographic images of the duodenoscope samples before and after incubation with the microbial strains and hydrogels. The samples are characterised using an inhomogeneous morphology, as has been previously observed in an optical study carried out on similar duodenoscopes [47]. AFM was also used to investigate whether biofilms were formed on the duodenoscope samples treated with hydrogel after incubation with *K. pneumoniae* (Figure 6k) and *E. faecalis* (Figure 6l). For both bacterial strains, no colonies or aggregates were found on the tested surfaces. In the case of *K. pneumoniae*, the attachment of rare rod-shaped cells was noticed. For *E. faecalis*, small round-shaped cells (with diameter of 0.7 μm) individually distributed on the solid surface were observed. Although the presence of few bacterial cells was confirmed, the bacteria left behind following hydrogel treatment failed to grow after incubation, suggesting that they had been destroyed by the hydrogels.

Additionally, from the SEM images, it can be noted that the hydrogels not only drastically reduced cell density and viability but also did not allow biofilm formation. It is known that, once formed, the presence of a biofilm on microorganisms protects them from outside influences, such as disinfectants and antibiotics [48]. A biofilm is an accumulation of microorganisms on a surface enclosed in a matrix of exopolysaccharides [49,50]. Additionally, it must be taken into account that there is a notable difference between “contamination”, meaning the presence of microorganisms on a duodenoscope, and “infection”, meaning microorganisms infiltrating human tissue or producing clinical disease; these are not synonymous. Duodenoscopes infected with specific microorganisms may result in infection. Not all organisms are harmful, and it is not always apparent how frequently they cause infections or what kind of damage they do. That being said, our results proved that generally, after 6 h of the incubation of the hydrogels, there was no chance of infection or contamination on the surface of the external polymers of the duodenoscope.

Our previous studies proved that iminoboronate–chitosan hydrogels based on 2-FPBA have fungicidal activity against various yeast strains such as *C. albicans*, *C. glabrata*, and *C. parapsilosis* [36,37]. Reports on formyl phenyl boronic acid (FPBA) showed its antimicrobial effects against *S. aureus*, *P. aeruginosa, Aspergillus* sp., *Fusarium* sp., *Penicillium* sp., and *Candida* sp. due to the presence of two hydroxyl groups [49,50,51]. In this particular case, BA was proven to destroy up to 85% of the *K. pneumoniae* cells and 75% of *C. albicans* cells after 6 h of incubation. BA was not very efficient against *E. faecalis*, destroying only 30% of the bacterial cells after 24 h of incubation.

Positive charges from the amino groups of chitosan interact electrostatically with negatively charged components on the microbial membrane, resulting in the disruption of cell membrane [52] or the blocking of the exchange of nutrients by covering porins [53], penetrating the cell wall to affect DNA/RNA and protein synthesis [54]. The antimicrobial effect of Ch depends on its molecular weight and varies among different microorganisms. This study proved that Ch destroys up to 90% of *K. pneumoniae* and up to 70% of *C. albicans* after 6 h of incubation. As in the case of BA, chitosan was not very efficient by itself against *E. faecalis*.

Usually, ZnO–NPs display antimicrobial activities via multiple mechanisms: interactions with the phospholipid bilayer, binding to cytosolic proteins, or the formation of reactive oxygen species (ROS), which produce damages to the phospholipids in the membranes, lipoproteins, and nucleic acids, causing oxidative stress and eventually destroying the microorganisms [55]. Additionally, the mechanism of ZnO–NPs implies the release of Zn^2+^ into the media, which causes bacterial damage through active transport inhibition, amino acid metabolism, and the disruption of an enzyme system [38]. The literature presents examples of ZnO–NPs with a higher antibacterial activity against Gram-negative strains than against Gram-positive strains [56,57,58]. Generally, it is thought that Gram-negative bacteria are more susceptible than Gram-positive to attack from external factors due to their cell wall composition [59]—in the case of Gram-negative bacteria, bacterial cells are covered by a layer of lipopolysaccharides (1–3 μm thick) and thin peptidoglycans (~8 nm thick), whereas Gram-positive bacteria have a peptidoglycan layer (~80 nm) thick) with covalently attached teichoic and teichuronic acids. Our results are similar to those presented in the literature, with ZnO–NPs being more efficient against *K. pneumoniae* (killing up to 90% of the bacterial cells after 24 h of incubation) than against *E. faecalis* (destroying up 20% of the bacterial cells after 24 h of incubation). ZnO–NPs also displayed limited antifungal activity, with 70% of cells being viable after the same period of incubation.

Recently, chitosan hybrid materials with metal NPs and oxide agents have been developed with the excellent properties of individual components and outstanding simultaneous synergistic effects [60]. Due to the presence of amine and hydroxyl groups on chitosan, the ZnO–chitosan complex is currently attracting great interest regarding its potential use as an antimicrobial agent [61]. The presence of ZnO–NPs in combination with chitosan as a natural compound that strongly complexes with metal oxide NPs has led to the increase in chitosan efficiency against *Campylobacter* sp., *M. luteus*, and *S. aureus* [61,62]. In this study, it was also noticed that ZnO–chitosan complex was very efficient against the tested reference strains after 6 h of incubation.

The antimicrobial activity determined for the ZnO–NPs is lower than that determined for the ZnO–chitosan complex, perhaps because chitosan acts as a barrier to the migration of ZnO to the medium and allows ZnO–NPs to be in direct contact with the bacterial cell surface, generating ROS and leading to cell death. The antimicrobial activity of the hydrogels is higher than that of the controls, and accumulates the activity of the precursors, their activity being significantly correlated with the incubation time.

## 4. Conclusions

In conclusion, both the structural and morphological features of the hydrogels contribute to their antimicrobial activity. The hydrogels presented very good antimicrobial activity against the different classes of microorganisms represented by a Gram-positive bacterial strain (*E. faecalis*), a Gram-negative bacterial strain (*K. pneumonia*), and a yeast strain (*C. albicans*). At the same time, the hydrogels limited the microbial adherence to the polymer coating of the duodenoscope. With these remarkable properties, the present hydrogels may be used in the prevention of bacterial biofilm development on duodenoscopes, as well as for the design of new antiseptics and disinfectants with efficient protective actions against the microorganism colonisation of inert surfaces.

## Figures and Tables

**Table 1:** The composition of the obtained systems.

Sample Code	m_chitosan_ (mg)	V_water_ (mL)	V_ac.acid_ (μL)	m_aldehyde_ (mg)	V_ethanol_ (mL)	NH_2_/CHO Molar Ratio	m_ZnO_ (mg)
S1	60	6	42	44.8	4.48	1	18
S1.5	60	6	42	33.3	3.33	1.5	18
S2	60	6	42	22.4	2.24	2	18
R	60	6	42	-	2.24	-	18

**Table 2:** Detailed statistics of the antimicrobial activity of the samples against: (a) *E. faecalis*; (b) *K. pneumoniae*; (c) *C. albicans*.

(a) Incubation Time	Relative Cell Viability (Mean ± SD)						
	Ch	BA	ZnO	R	S1	S1.5	S2
10 min	42.10 ± 0.22 ***	99.48 ± 0.68 ***	99.75 ± 0.28 ***	72.25 ± 2.01 ***	16.98 ± 1.37 ***	10.60 ± 0.50 ***	27.36 ± 0.99 ***
20 min	24.89 ± 1.01 ***	98.58 ± 0.94 ***	99.66 ± 0.58 ***	9.45 ± 0.86 ***	8.43 ± 0.70 ***	7.99 ± 0.40 ***	18.43 ± 0.70 ***
40 min	9.32 ± 0.71 ***	95.23 ± 1.06 ***	86.96 ± 2.76 ***	3.08 ± 0.95 ***	4.70 ± 0.55 ***	1.52 ± 0.26 ***	14.52 ± 0.86 ***
1 h	11.61 ± 0.91 ***	86.82 ± 1.52 ***	79.45 ± 0.87 ***	2.82 ± 0.34 ***	4.46 ± 0.60 ***	1.17 ± 0.26 ***	8.02 ± 0.27 ***
2 h	13.23 ± 0.22 ***	90.94 ± 3.29 ***	82.42 ± 1.04 ***	2.11 ± 0.10 ***	0.66 ± 0.20 ***	0.00 ± 0.00 ***	4.34 ± 0.67 ***
3 h	12.58 ± 0.64 ***	75.84 ± 1.58 ***	88.48 ± 1.12 ***	2.04 ± 0.22 ***	0.23 ± 0.24 ***	0.00 ± 0.00 ***	2.00 ± 0.13 ***
6 h	4.49 ± 0.60 ***	76.87 ± 2.83 ***	87.78 ± 0.75 ***	0.66 ± 0.21 ***	0.00 ± 0.00 ***	0.00 ± 0.00 ***	0.00 ± 0.00 ***
24 h	0.49 ± 0.29 ***	70.59 ± 1.12 ***	79.63 ± 0.74 ***	0.00 ± 0.00 ***	0.00 ± 0.00 ***	0.00 ± 0.00 ***	0.00 ± 0.00 ***
**(b)** **Incubation Time**	**Relative Cell Viability (Mean ± SD)**						
	**Ch**	**BA**	**ZnO**	**R**	**S1**	**S1.5**	**S2**
10 min	86.66 ± 3.16 ***	99.75 ± 0.30 ***	94.95 ± 1.72 ***	67.70 ± 1.13 ***	86.71 ± 3.39 ***	71.28 ± 1.26 ***	87.30 ± 1.47 ***
20 min	84.19 ± 0.86 ***	97.63 ± 1.06 ***	97.96 ± 2.54 ***	64.89 ± 1.56 ***	84.79 ± 0.58 ***	60.36 ± 0.97 ***	79.27 ± 0.88 ***
40 min	79.51 ± 0.97 ***	87.73 ± 0.90 ***	98.09 ± 0.35 ***	39.62 ± 1.01 ***	13.62 ± 0.85 ***	22.62 ± 1.26 ***	42.56 ± 2.06 ***
1 h	57.73 ± 1.99 ***	84.13 ± 1.35 ***	95.93 ± 0.70 ***	26.42 ± 1.18 ***	9.66 ± 0.41 ***	11.43 ± 0.84 ***	42.23 ± 2.86 ***
2 h	28.08 ± 1.77 ***	40.48 ± 0.80 ***	93.23 ± 2.89 ***	13.09 ± 0.65 ***	8.12 ± 0.79 ***	9.40 ± 0.67 ***	24.64 ± 1.01 ***
3 h	25.10 ± 0.97 ***	25.26 ± 0.46 ***	69.44 ± 1.19 ***	11.30 ± 1.21 ***	5.23 ± 1.21 ***	7.73 ± 1.02 ***	19.11 ± 0.87 ***
6 h	9.49 ± 0.88 ***	18.28 ± 1.70 ***	8.96 ± 0.22 ***	1.18 ± 0.29 ***	0.00 ± 0.00 ***	0.07 ± 0.12 ***	1.50 ± 0.45 ***
24 h	0.21 ± 0.11 ***	4.96 ± 0.48 ***	1.84 ± 0.65 ***	0.00 ± 0.00 ***	0.00 ± 0.00 ***	0.00 ± 0.00 ***	0.00 ± 0.00 ***
**(c)** **Incubation Time**	**Relative Cell Viability (Mean ± SD)**						
	**Ch**	**BA**	**ZnO**	**R**	**S1**	**S1.5**	**S2**
10 min	86.92 ± 1.60 ***	79.82 ± 2.49 ***	93.34 ± 0.95 ***	99.52 ± 0.70 ***	72.57 ± 1.34 ***	63.34 ± 2.95 ***	85.31 ± 4.89 ***
20 min	48.53 ± 1.52 ***	77.98 ± 2.54 ***	92.54 ± 1.58 ***	93.54 ± 1.70 ***	44.56 ± 0.97 ***	60.20 ± 1.13 ***	93.13 ± 3.36 ***
40 min	44.51 ± 3.67 ***	79.57 ± 1.48 ***	92.13 ± 2.63 ***	74.13 ± 0.31 ***	39.27 ± 0.83 ***	59.54 ± 1.25 ***	91.20 ± 1.24 ***
1 h	42.12 ± 4.29 ***	82.80 ± 2.51 ***	76.14 ± 2.30 ***	71.24 ± 1.39 ***	45.93 ± 2.45 ***	58.33 ± 2.17 ***	88.38 ± 1.41 ***
2 h	45.54 ± 5.62 ***	70.99 ± 0.74 ***	70.36 ± 0.68 ***	55.44 ± 1.14 ***	40.30 ± 0.91 ***	56.38 ± 3.48 ***	78.29 ± 2.55 ***
3 h	48.35 ± 2.79 ***	65.36 ± 4.83 ***	72.54 ± 2.50 ***	48.34 ± 1.18 ***	38.67 ± 1.82 ***	41.35 ± 1.06 ***	69.90 ± 0.20 ***
6 h	28.53 ± 2.25 ***	34.21 ± 0.93 ***	70.57 ± 0.76 ***	35.52 ± 2.42 ***	38.90 ± 3.65 ***	34.66 ± 0.65 ***	34.47 ± 3.95 ***
24 h	23.36 ± 2.93 ***	0.00 ± 0.00 ***	68.22 ± 2.00 ***	0.00 ± 0.00 ***	0.00 ± 0.00 ***	0.00 ± 0.00 ***	0.00 ± 0.00 ***

*** *p* < 0.0001.

## Data Availability

The data presented in this study are available on request from the corresponding author.

## References

[1] Larsen S., Russell R.V., Ockert L.K., Spanos S., Travis H.S., Ehlers L.H., Mærkedahl A. (2020). Rate and Impact of Duodenoscope Contamination: A Systematic Review and Meta-Analysis. EClinicalMedicine.

[2] Adler D.G., Lieb J.G., Cohen J., Pike I.M., Park W.G., Rizk M.K., Sawhney M.S., Scheiman J.M., Shaheen N.J., Sherman S. (2015). Quality Indicators for ERCP. Gastrointest. Endosc..

[3] Okamoto N., Sczaniecka A., Hirano M., Benedict M., Baba S., Horino Y., Takenouchi M., Morizane Y., Yana I., Segan R. (2022). A Prospective, Multicenter, Clinical Study of Duodenoscope Contamination after Reprocessing. Infect. Control Hosp. Epidemiol.

[4] Ribeiro M.M., de Oliveira A.C., Ribeiro S.M.C.P., Watanabe E., de Resende Stoianoff M.A., Ferreira J.A.G. (2013). Effectiveness of Flexible Gastrointestinal Endoscope Reprocessing. Infect. Control Hosp. Epidemiol..

[5] Kovaleva J. (2016). Infectious Complications in Gastrointestinal Endoscopy and Their Prevention. Best Pract. Res. Clin. Gastroenterol..

[6] Visrodia K.H., Ofstead C.L., Yellin H.L., Wetzler H.P., Tosh P.K., Baron T.H. (2014). The Use of Rapid Indicators for the Detection of Organic Residues on Clinically Used Gastrointestinal Endoscopes with and without Visually Apparent Debris. Infect Control Hosp. Epidemiol..

[7] Ross A.S., Baliga C., Verma P., Duchin J., Gluck M. (2015). A Quarantine Process for the Resolution of Duodenoscope-Associated Transmission of Multidrug-Resistant Escherichia Coli. Gastrointest. Endosc..

[8] Chapman C.G., Siddiqui U.D., Manzano M., Konda V.J., Murillo C., Landon E.M., Waxman I. (2017). Risk of Infection Transmission in Curvilinear Array Echoendoscopes: Results of a Prospective Reprocessing and Culture Registry. Gastrointest. Endosc..

[9] Rauwers A.W., Voor In ’t Holt A.F., Buijs J.G., de Groot W., Hansen B.E., Bruno M.J., Vos M.C. (2018). High Prevalence Rate of Digestive Tract Bacteria in Duodenoscopes: A Nationwide Study. Gut.

[10] Balan G.G., Sfarti C.V., Chiriac S.A., Stanciu C., Trifan A. (2019). Duodenoscope-Associated Infections: A Review. Eur. J. Clin. Microbiol. Infect Dis..

[11] Mark J.A., Underberg K., Kramer R.E. (2020). Results of Duodenoscope Culture and Quarantine after Manufacturer-Recommended Cleaning Process. Gastrointest. Endosc..

[12] Heuvelmans M., Wunderink H.F., van der Mei H.C., Monkelbaan J.F. (2021). A Narrative Review on Current Duodenoscope Reprocessing Techniques and Novel Developments. Antimicrob. Resist. Infect. Control.

[13] Kwakman J.A., Bexkens M.L., Bruno M.J., Vos M.C. (2022). Persistent Contamination of a Duodenoscope Working Channel in a Non-Clinical Simulated ERCP Setting. Endoscopy.

[14] Bajolet O., Ciocan D., Vallet C., de Champs C., Vernet-Garnier V., Guillard T., Brasme L., Thiefin G., Cadiot G., Bureau-Chalot F. (2013). Gastroscopy-Associated Transmission of Extended-Spectrum Beta-Lactamase-Producing Pseudomonas Aeruginosa. J. Hosp. Infect..

[15] Epstein L., Hunter J.C., Arwady M.A., Tsai V., Stein L., Gribogiannis M., Frias M., Guh A.Y., Laufer A.S., Black S. (2014). New Delhi Metallo-β-Lactamase–Producing Carbapenem-Resistant Escherichia Coli Associated with Exposure to Duodenoscopes. JAMA.

[16] Smith Z.L., Oh Y.S., Saeian K., Edmiston C.E., Khan A.H., Massey B.T., Dua K.S. (2015). Transmission of Carbapenem-Resistant Enterobacteriaceae during ERCP: Time to Revisit the Current Reprocessing Guidelines. Gastrointest. Endosc..

[17] Yang S., Hemarajata P., Hindler J., Li F., Adisetiyo H., Aldrovandi G., Sebra R., Kasarskis A., MacCannell D., Didelot X. (2017). Evolution and Transmission of Carbapenem-Resistant Klebsiella Pneumoniae Expressing the BlaOXA-232 Gene During an Institutional Outbreak Associated With Endoscopic Retrograde Cholangiopancreatography. Clin. Infect. Dis..

[18] Bourigault C., Le Gallou F., Bodet N., Musquer N., Juvin M.-E., Corvec S., Ferronnière N., Wiesel S., Gournay J., Birgand G. (2018). Duodenoscopy: An Amplifier of Cross-Transmission during a Carbapenemase-Producing Enterobacteriaceae Outbreak in a Gastroenterology Pathway. J. Hosp. Infect..

[19] Bălan G.G., Roșca I., Ursu E.-L., Doroftei F., Bostănaru A.-C., Hnatiuc E., Năstasă V., Șandru V., Ștefănescu G., Trifan A. (2018). Plasma-Activated Water: A New and Effective Alternative for Duodenoscope Reprocessing. IDR.

[20] Balan G.G., Rosca I., Ursu E.-L., Fifere A., Varganici C.-D., Doroftei F., Turin-Moleavin I.-A., Sandru V., Constantinescu G., Timofte D. (2019). Duodenoscope-Associated Infections beyond the Elevator Channel: Alternative Causes for Difficult Reprocessing. Molecules.

[21] Liu Y., He L., Mustapha A., Li H., Hu Z.q., Lin M. (2009). Antibacterial Activities of Zinc Oxide Nanoparticles against Escherichia Coli O157:H7. J. Appl. Microbiol..

[22] Narayanan P.M., Wilson W.S., Abraham A.T., Sevanan M. (2012). Synthesis, Characterization, and Antimicrobial Activity of Zinc Oxide Nanoparticles Against Human Pathogens. BioNanoScience.

[23] Espitia P.J.P., de Soares F.F., dos Reis Coimbra J.S., de Andrade N.J., Cruz R.S., Medeiros E.A.A. (2012). Zinc Oxide Nanoparticles: Synthesis, Antimicrobial Activity and Food Packaging Applications. Food Bioprocess Technol..

[24] Watson C.Y., Molina R.M., Louzada A., Murdaugh K.M., Donaghey T.C., Brain J.D. (2015). Effects of Zinc Oxide Nanoparticles on Kupffer Cell Phagosomal Motility, Bacterial Clearance, and Liver Function. IJN.

[25] Yazdanshenas M.E., Shateri-Khalilabad M. (2013). In Situ Synthesis of Silver Nanoparticles on Alkali-Treated Cotton Fabrics. J. Ind. Text..

[26] Jesline A., John N.P., Narayanan P.M., Vani C., Murugan S. (2015). Antimicrobial Activity of Zinc and Titanium Dioxide Nanoparticles against Biofilm-Producing Methicillin-Resistant Staphylococcus Aureus. Appl. Nanosci..

[27] Reddy L.S., Nisha M.M., Joice M., Shilpa P.N. (2014). Antimicrobial Activity of Zinc Oxide (ZnO) Nanoparticle against Klebsiella Pneumoniae. Pharm. Biol..

[28] Quek J.-A., Lam S.-M., Sin J.-C., Mohamed A.R. (2018). Visible Light Responsive Flower-like ZnO in Photocatalytic Antibacterial Mechanism towards Enterococcus Faecalis and Micrococcus Luteus. J. Photochem. Photobiol. B Biol..

[29] Mirhosseini F., Amiri M., Daneshkazemi A., Zandi H., Javadi Z.S. (2019). Antimicrobial Effect of Different Sizes of Nano Zinc Oxide on Oral Microorganisms. Front. Dent..

[30] Jie L.J., Chi L.Z., Wong L.S., Rajamani R., Djearamane S. (2022). Antibacterial Efficacy of Zinc Oxide Nanoparticles against Serratia Marcescens (ATCC 43862) and Enterococcus Faecalis (ATCC 29121). J. Exp. Biol. Agric. Sci..

[31] Djearamane S., Loh Z.C., Lee J.J., Wong L.S., Rajamani R., Luque P.A., Gupta P.K., Liang S.X.T. (2022). Remedial Aspect of Zinc Oxide Nanoparticles Against Serratia Marcescens and Enterococcus Faecalis. Front. Pharmacol..

[32] Sharma R.K., Ghose R. (2015). Synthesis of Zinc Oxide Nanoparticles by Homogeneous Precipitation Method and Its Application in Antifungal Activity against Candida Albicans. Ceram. Int..

[33] Sharma D.K., Shukla S., Sharma K.K., Kumar V. (2022). A Review on ZnO: Fundamental Properties and Applications. Mater. Today Proc..

[34] Huh A.J., Kwon Y.J. (2011). “Nanoantibiotics”: A New Paradigm for Treating Infectious Diseases Using Nanomaterials in the Antibiotics Resistant Era. J. Control. Release.

[35] Slavin Y.N., Asnis J., Häfeli U.O., Bach H. (2017). Metal Nanoparticles: Understanding the Mechanisms behind Antibacterial Activity. J. Nanobiotechnol..

[36] Ailincai D., Marin L., Morariu S., Mares M., Bostanaru A.-C., Pinteala M., Simionescu B.C., Barboiu M. (2016). Dual Crosslinked Iminoboronate-Chitosan Hydrogels with Strong Antifungal Activity against Candida Planktonic Yeasts and Biofilms. Carbohydr. Polym..

[37] Ailincai D., Rosca I., Morariu S., Mititelu-Tartau L., Marin L. (2022). Iminoboronate-Chitooligosaccharides Hydrogels with Strong Antimicrobial Activity for Biomedical Applications. Carbohydr. Polym..

[38] Sirelkhatim A., Mahmud S., Seeni A., Kaus N.H.M., Ann L.C., Bakhori S.K.M., Hasan H., Mohamad D. (2015). Review on Zinc Oxide Nanoparticles: Antibacterial Activity and Toxicity Mechanism. Nano-Micro Lett..

[39] Nemecek T., Komanec M., Nelsen B., Martan T., Suslov D., Hartmann P., Zvanovec S. (2018). Experimentally and Analytically Derived Generalized Model for the Detection of Liquids with Suspended-Core Optical Fibers. Opt. Fiber Technol..

[40] Zhou Z., Yan D., Cheng X., Kong M., Liu Y., Feng C., Chen X. (2016). Biomaterials Based on N,N,N-Trimethyl Chitosan Fibers in Wound Dressing Applications. Int. J. Biol. Macromol..

[41] Ailincai D., Bercea M., Mititelu Tartau L., Marin L. (2022). Biocompatible Drug Delivery Systems Able to Co-Deliver Antifungal and Antiviral Agents. Carbohydr. Polym..

[42] Ailincai D., Rosca I. (2023). New Hydrogels and Formulations Based on Piperonyl-Imino-Chitosan Derivatives. Polymers.

[43] Rajendran S.P., Sengodan K. (2017). Synthesis and Characterization of Zinc Oxide and Iron Oxide Nanoparticles Using *Sesbania Grandiflora* Leaf Extract as Reducing Agent. J. Nanosci..

[44] Marin L., Moraru S., Popescu M.-C., Nicolescu A., Zgardan C., Simionescu B.C., Barboiu M. (2014). Out-of-Water Constitutional Self-Organization of Chitosan–Cinnamaldehyde Dynagels. Chem. A Eur. J..

[45] Talam S., Karumuri S.R., Gunnam N. (2012). Synthesis, Characterization, and Spectroscopic Properties of ZnO Nanoparticles. Int. Sch. Res. Not..

[46] Ghamsari M.S., Alamdari S., Razzaghi D., Arshadi Pirlar M. (2019). ZnO Nanocrystals with Narrow-Band Blue Emission. J. Lumin..

[47] Balan G.G., Pavel L., Sandu A.V., Stefanescu G., Trifan A.V. (2016). Preliminary Study on Erosion of Polymer Coatings of Duodenoscopes. Mater. Plast..

[48] Verfaillie C.J., Bruno M.J., Voor in ’t Holt A.F., Buijs J.G., Poley J.-W., Loeve A.J., Severin J.A., Abel L.F., Smit B.J., de Goeij I. (2015). Withdrawal of a Novel-Design Duodenoscope Ends Outbreak of a VIM-2-Producing Pseudomonas Aeruginosa. Endoscopy.

[49] Adamczyk-Woźniak A., Gozdalik J.T., Wieczorek D., Madura I.D., Kaczorowska E., Brzezińska E., Sporzyński A., Lipok J. (2020). Synthesis, Properties and Antimicrobial Activity of 5-Trifluoromethyl-2-Formylphenylboronic Acid. Molecules.

[50] Abid H.M.U., Hanif M., Mahmood K., Aziz M., Abbas G., Latif H. (2022). Wound-Healing and Antibacterial Activity of the Quercetin–4-Formyl Phenyl Boronic Acid Complex against Bacterial Pathogens of Diabetic Foot Ulcer. ACS Omega.

[51] Borys K.M., Wieczorek D., Pecura K., Lipok J., Adamczyk-Woźniak A. (2019). Antifungal Activity and Tautomeric Cyclization Equilibria of Formylphenylboronic Acids. Bioorganic Chem..

[52] Matica M.A., Aachmann F.L., Tøndervik A., Sletta H., Ostafe V. (2019). Chitosan as a Wound Dressing Starting Material: Antimicrobial Properties and Mode of Action. Int. J. Mol. Sci..

[53] Feng P., Luo Y., Ke C., Qiu H., Wang W., Zhu Y., Hou R., Xu L., Wu S. (2021). Chitosan-Based Functional Materials for Skin Wound Repair: Mechanisms and Applications. Front. Bioeng. Biotechnol..

[54] Ke C.-L., Deng F.-S., Chuang C.-Y., Lin C.-H. (2021). Antimicrobial Actions and Applications of Chitosan. Polymer.

[55] Raghunath A., Perumal E. (2017). Metal Oxide Nanoparticles as Antimicrobial Agents: A Promise for the Future. Int. J. Antimicrob. Agents.

[56] Petkova P., Francesko A., Fernandes M.M., Mendoza E., Perelshtein I., Gedanken A., Tzanov T. (2014). Sonochemical Coating of Textiles with Hybrid ZnO/Chitosan Antimicrobial Nanoparticles. ACS Appl. Mater. Interfaces.

[57] Sathiya S.M., Okram G.S., Maria Dhivya S., Manivannan G., Jothi Rajan M.A. (2016). Interaction of Chitosan/Zinc Oxide Nanocomposites and Their Antibacterial Activities with Escherichia Coli. Mater. Today Proc..

[58] Barreto M.S., Andrade C.T., Azero E.G., Paschoalin V.M., Del Aguila E.M. (2017). Production of Chitosan/Zinc Oxide Complex by Ultrasonic Treatment with Antibacterial Activity. J. Bacteriol. Parasitol..

[59] Piktel E., Suprewicz Ł., Depciuch J., Chmielewska S., Skłodowski K., Daniluk T., Król G., Kołat-Brodecka P., Bijak P., Pajor-Świerzy A. (2021). Varied-Shaped Gold Nanoparticles with Nanogram Killing Efficiency as Potential Antimicrobial Surface Coatings for the Medical Devices. Sci. Rep..

[60] Li L.-H., Deng J.-C., Deng H.-R., Liu Z.-L., Xin L. (2010). Synthesis and Characterization of Chitosan/ZnO Nanoparticle Composite Membranes. Carbohydr. Res..

[61] Dhillon G.S., Kaur S., Brar S.K. (2014). Facile Fabrication and Characterization of Chitosan-Based Zinc Oxide Nanoparticles and Evaluation of Their Antimicrobial and Antibiofilm Activity. Int. Nano Lett..

[62] Mohammed A.N., Abdel Aziz S.A.A. (2019). The Prevalence of Campylobacter Species in Broiler Flocks and Their Environment: Assessing the Efficiency of Chitosan/Zinc Oxide Nanocomposite for Adopting Control Strategy. Environ. Sci. Pollut. Res..

